# Pseudotyping of VSV with Ebola virus glycoprotein is superior to HIV-1 for the assessment of neutralising antibodies

**DOI:** 10.1038/s41598-020-71225-1

**Published:** 2020-08-31

**Authors:** Kimberley Steeds, Yper Hall, Gillian S. Slack, Stephanie Longet, Thomas Strecker, Sarah Katharina Fehling, Edward Wright, Joseph Akoi Bore, Fara Raymond Koundouno, Mandy Kader Konde, Roger Hewson, Julian A. Hiscox, Georgios Pollakis, Miles W. Carroll

**Affiliations:** 1 Public Health England (PHE), Porton Down, Salisbury, Wiltshire UK; 2 Institute of Virology, Philipps University Marburg, Marburg, Germany; 3 School of Life Sciences, University of Sussex, Brighton, UK; 4 Institut National de Santé Publique, Conakry, Republic of Guinea; 5 University Julius Nyerere of Kankan, Conakry, Republic of Guinea; 6 Centre d’Excellence de Formation et Recherche sur le Paludisme et les Maladies Prioritaires en Guinée (CEFORPAG), Ratoma, Conakry, Republic of Guinea; 7 Institute of Infection and Global Health (IGH), University of Liverpool, Liverpool, UK

**Keywords:** Immunology, Diseases, Virology

## Abstract

Ebola virus (EBOV) is an enveloped, single-stranded RNA virus that can cause Ebola virus disease (EVD). It is thought that EVD survivors are protected against subsequent infection with EBOV and that neutralising antibodies to the viral surface glycoprotein (GP) are potential correlates of protection. Serological studies are vital to assess neutralising antibodies targeted to EBOV GP; however, handling of EBOV is limited to containment level 4 laboratories. Pseudotyped viruses can be used as alternatives to live viruses, which require high levels of bio-containment, in serological and viral entry assays. However, neutralisation capacity can differ among pseudotyped virus platforms. We evaluated the suitability of EBOV GP pseudotyped human immunodeficiency virus type 1 (HIV-1) and vesicular stomatitis virus (VSV) to measure the neutralising ability of plasma from EVD survivors, when compared to results from a live EBOV neutralisation assay. The sensitivity, specificity and correlation with live EBOV neutralisation were greater for the VSV-based pseudotyped virus system, which is particularly important when evaluating EBOV vaccine responses and immuno-therapeutics. Therefore, the EBOV GP pseudotyped VSV neutralisation assay reported here could be used to provide a better understanding of the putative correlates of protection against EBOV.

## Introduction

Ebola virus (EBOV), a member of the family *Filoviridae*, is an enveloped, single-stranded RNA virus that can cause Ebola virus disease (EVD), a highly lethal illness with up to 90% mortality^1^. Since its discovery in 1976, EBOV has caused sporadic outbreaks across Central Africa and was responsible for the 2013–2016 EVD epidemic in West Africa^2^, which was the largest EBOV outbreak on record and resulted in more than 28,600 cases and over 11,300 deaths^3^. This outbreak constituted a public health emergency of international concern and highlighted the urgent need for vaccines and therapeutics against EBOV.

The EBOV RNA genome is approximately 19 kb in length and encodes seven main proteins. The envelope glycoprotein (GP) of EBOV forms homotrimeric spikes that project from the surface of the viral particles^4^. Surface GP is critical for host cell attachment and fusion^5,6^ and is a target for neutralising antibodies^7^. Survivors of EVD are thought to protected against subsequent EBOV infection and neutralising antibodies to the viral surface GP are possible correlates of protection^8,9^. Serological assays, such as the plaque reduction neutralisation test (PRNT), are central to evaluate neutralising antibodies against EBOV GP. However, because of its severe pathogenicity, potential transmission from person-to-person and lack of approved vaccines or antiviral treatments, handling of EBOV is restricted to containment level (CL) 4 laboratories. High containment facilities are expensive and are not readily available, especially in countries and organisations with limited resources. Furthermore, the assay format and time required for plaque development, which can take approximately nine days, makes it time-consuming and restricts high-throughput sample processing. Development of novel serological assays that utilise genetically modified recombinant or chimeric viruses with attenuated pathogenicity have enabled more widespread investigation of neutralising antibodies against highly pathogenic viruses including EBOV^10,11^.

Pseudotyped viruses are replication-defective chimeric virions that comprise the structural and enzymatic core of one virus, bearing the envelope protein or glycoprotein of another, and encode a quantifiable reporter gene. Retroviruses, including lentiviruses and gammaretroviruses such as human immunodeficiency virus (HIV) and murine leukaemia virus (MLV), respectively, and rhabdoviruses, such as vesicular stomatitis virus (VSV), have been used extensively as cores for pseudotyped viruses^12,13^, including for EBOV^14,15^. A number of EBOV GP pseudotyped virus neutralisation assays have been developed to investigate immune responses to EBOV infection and vaccination^16–18^, as well as for evaluation of monoclonal antibody (mAb) therapies^19–21^.

There are many factors that need to be considered when developing and optimising pseudotyped virus neutralisation assays, to assess experimental parameters that can affect assay performance and to ensure accuracy and reproducibility. These include, choice of core virus and reporter gene, determination of target cell line and amount of pseudotyped virus input, as well as correlation with live virus neutralisation^22^. The aim of this study was to assess the suitability of EBOV GP pseudotyped HIV-1 and VSV systems to measure neutralisation by EVD survivor plasma, in comparison with results from a live EBOV neutralisation assay.

## Results

### Cell tropism of EBOV GP pseudotyped viruses

**Figure 1:**
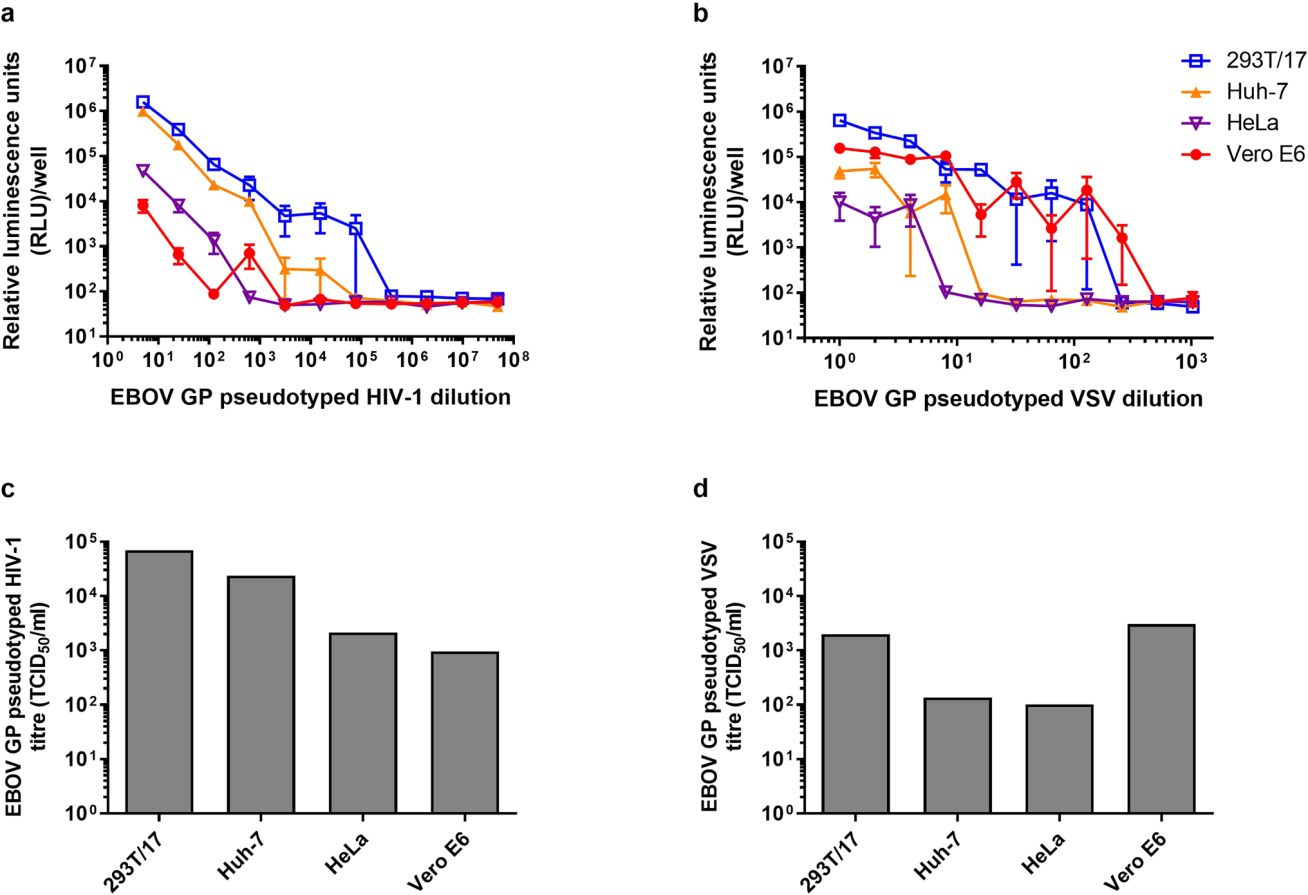
Titration of EBOV (Mayinga) GP pseudotyped (**a**) HIV-1 and (**b**) VSV using different cell lines. Relative luminescence units per well (RLU/well) were measured. Error bars are one standard error above and below the mean, n = 4. EBOV GP pseudotyped (**c**) HIV-1 and (**d**) VSV titres expressed as 50% tissue culture infectious dose per ml (TCID_50_/ml), n = 1.

Pseudotyped HIV-1 and VSV bearing the envelope GP from EBOV (Mayinga) were generated and quantified by measuring luminescence in a range of target cell lines, in order to determine the optimum cell line to use in neutralisation assays. Cells only controls were used to determine background levels of luminescence (Supplementary Fig. S1). Reporter activity was detected in all cell lines infected with EBOV GP pseudotyped HIV-1 and VSV, demonstrating the broad tissue range conferred by EBOV GP, although differences in luminescence were observed (Fig. 1a,b). For EBOV GP pseudotyped HIV-1, highest TCID_50_/ml values were observed in 293T/17 cells, followed by Huh-7 cells (Fig. 1c). Titres generated by infection of 293T/17 cells were approximately 3, 33 and 73 times greater than those produced by infection of Huh-7, HeLa and Vero E6 cells, respectively. For EBOV GP pseudotyped VSV, highest titres were obtained in Vero E6 cells. The TCID_50_/ml values generated by infection of Vero E6 cells were approximately 1.5, 22 and 30 times greater than those produced by infection of 293T/17, Huh-7 and HeLa cells, respectively (Fig. 1d). Based on these results, the 293T/17 and Vero E6 target cell lines were selected for use in all subsequent neutralisation assays using EBOV GP pseudotyped HIV-1 and VSV, respectively.

### Neutralisation of EBOV GP pseudotyped viruses by anti-EBOV GP mAb

**Figure 2:**
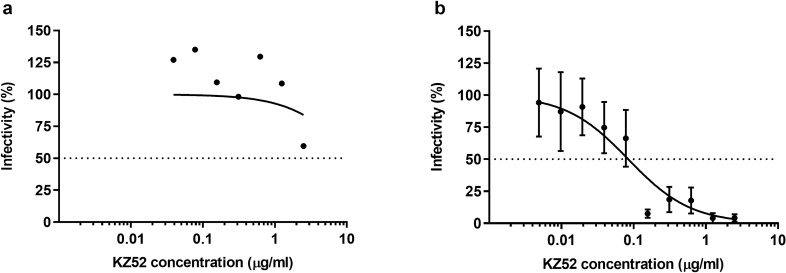
Neutralisation of EBOV (Mayinga) GP pseudotyped (**a**) HIV-1, n = 2, and (**b**) VSV, n = 4, by human anti-EBOV GP mAb, KZ52. Percentage infectivity was calculated relative to pseudotyped virus only controls. Data are shown for mean with log (inhibitor) vs. normalised response curves. Error bars are 1 standard error above and below the mean. Dotted lines represent 50% infectivity.

During the initial stages of assay development, it is important to evaluate neutralisation of pseudotyped viruses using well characterised antibodies in order to demonstrate the validity and accuracy of the assay. The EBOV GP pseudotyped viruses were assessed for neutralisation by the human anti-EBOV GP mAb, KZ52. KZ52 is an antibody isolated from a human survivor of the 1995 outbreak in Kikwit that neutralises EBOV in vitro and recognises a conformational epitope at the base of the GP^23–25^. Human anti-EBOV GP mAb, KZ52 was unable to neutralise the EBOV GP pseudotyped HIV-1 (Fig. 2a) within the range tested, however it was able to neutralise the EBOV GP pseudotyped VSV (Fig. 2b), suggesting that VSV-based pseudotyped viruses are more sensitive to neutralisation then lentiviral-based, possibly the density of EBOV GP on the pseudotyped HIV-1 may differ from that on the pseudotyped VSV or live EBOV.

### Assessment of EBOV GP pseudotyped virus input for neutralisation

**Figure 3:**
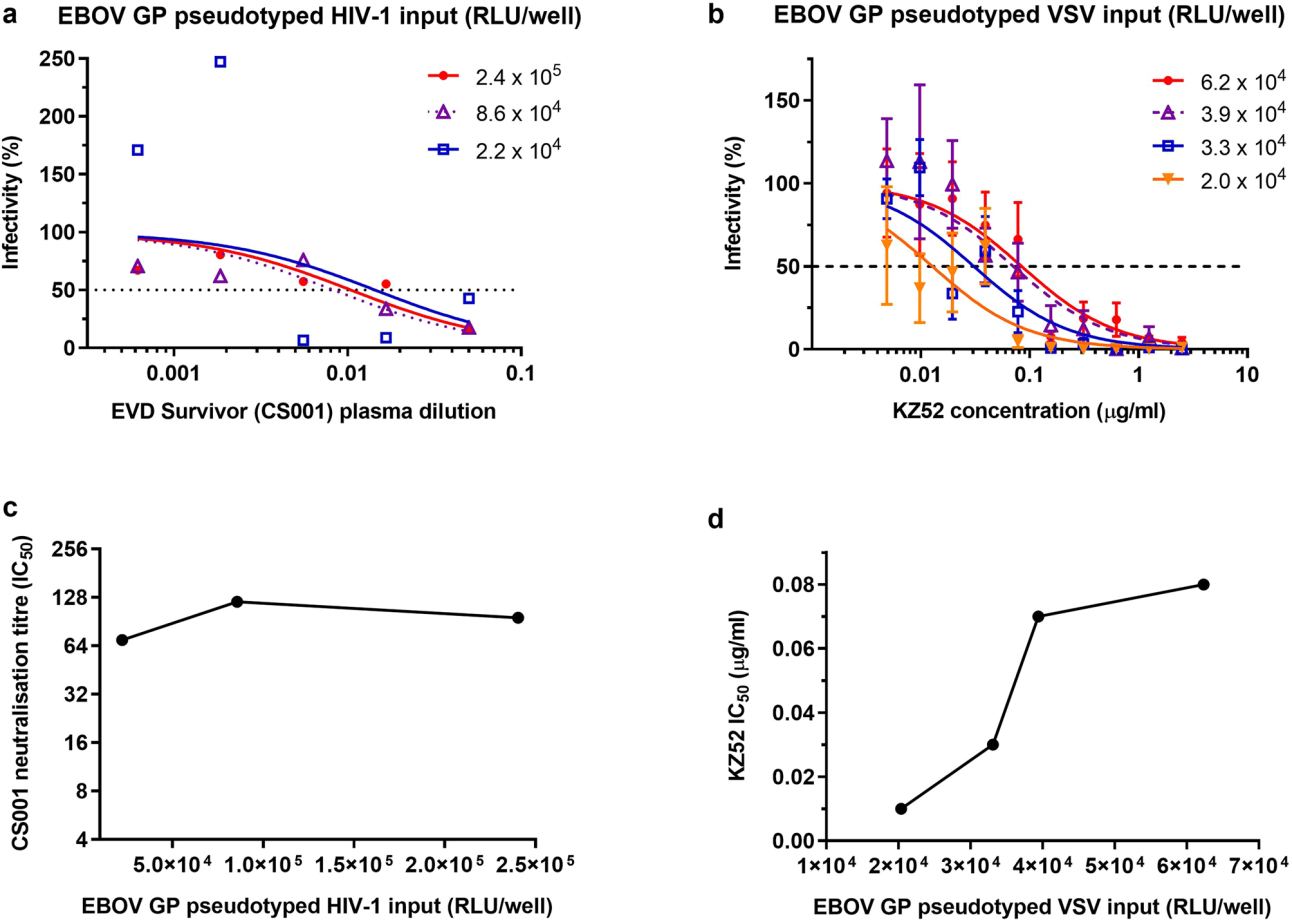
Effect of EBOV (Mayinga) GP pseudotyped (**a**) HIV-1 input on neutralisation by EVD survivor (CS001) plasma, n = 2, and (**b**) VSV by anti-EBOV GP mAb, KZ52, n = 4. Percentage infectivity was calculated relative to pseudotyped virus only controls. Data are shown for mean with log (inhibitor) vs. normalised response curves. Error bars are 1 standard error above and below the mean. Dotted lines represent 50% infectivity. The IC_50_ of EBOV GP pseudotyped (**c**) HIV-1 and (**d**) VSV neutralisation were estimated by model of nonlinear regression dose–response curves, n = 1.

To determine the optimal pseudotyped virus input to use in the HIV- and VSV-based assays, neutralisation of different amounts of the EBOV GP pseudotyped viruses by plasma from a Guinean EVD survivor donor or human anti-EBOV GP mAb KZ52 was assessed. KZ52 was selected as it is commercially available and there is accompanying information regarding its neutralisation activity against EBOV GP pseudotyped VSV expressing luciferase. However, as the EBOV GP pseudotyped HIV-1 was not neutralised by KZ52 (Fig. 2a) in the range tested, plasma from an EVD survivor was used to assess the effect of pseudotyped HIV-1 input on neutralisation instead. Survivor plasma sample CS001 was chosen as it displayed strong neutralising ability against live EBOV neutralisation, with a geometric mean titre (GMT) of 1,218. Percentage infectivity was determined relative to infectivity of cells by the EBOV GP pseudotyped viruses alone (Fig. 3a,b) and 50% inhibitory concentration (IC_50_) of pseudotyped virus neutralisation were estimated by model of nonlinear regression dose–response curves (Fig. 3c,d). Plasma from EVD survivor CS001 displayed neutralising activity against all amounts of EBOV GP pseudotyped HIV-1 tested (Fig. 3a). Lower pseudotyped virus input resulted in larger variability and less curve fitting. Therefore, an EBOV GP pseudotyped HIV-1 input of at least 8.6 × 10^4^ RLU/well, with a target input of 2.0 × 10^5^ RLU/well, was used in subsequent neutralisation assays. KZ52 neutralised all dilutions of EBOV GP pseudotyped VSV tested (Fig. 3b) and IC_50_ values decreased with decreasing amounts of pseudotyped virus input (Fig. 3d, Supplementary Table S1). When using 3.9 × 10^4^ RLU/well of EBOV GP pseudotyped VSV, IC_50_ of virus neutralisation (0.07 µg/ml) was similar to that expected according to the manufacturer’s product data sheet (0.06 µg/ml). Therefore, a target input of approximately 3.7 × 10^4^ RLU/well was used in subsequent EBOV GP pseudotyped VSV neutralisation assays.

### Neutralisation of EBOV GP pseudotyped viruses by EVD survivor plasma

**Figure 4:**
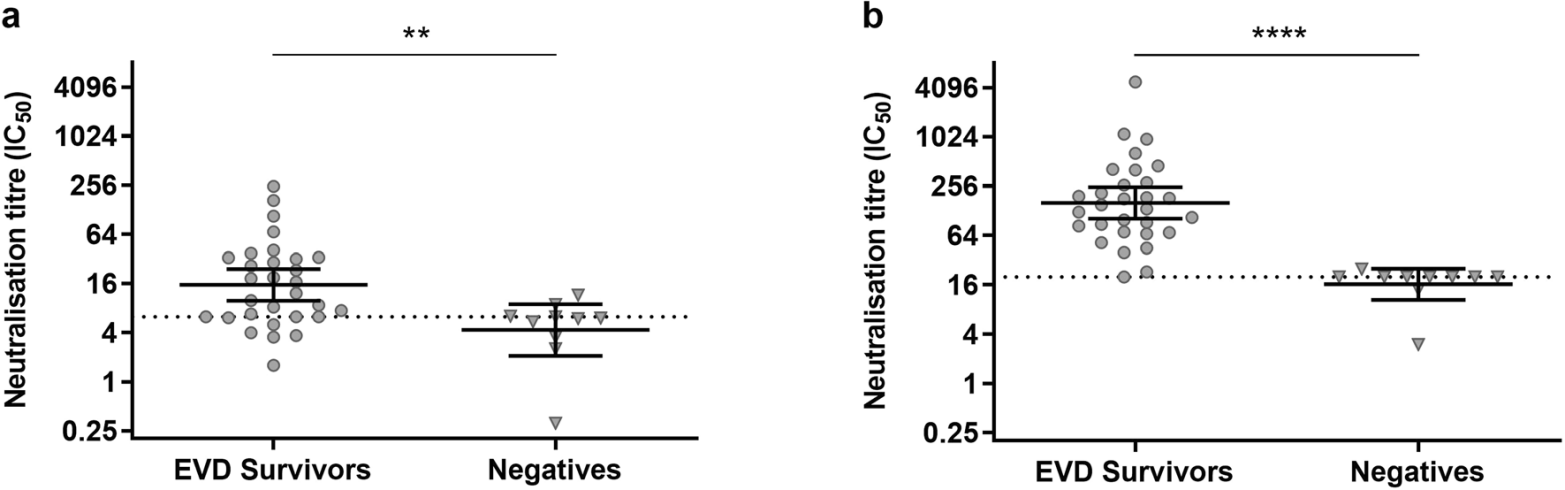
Neutralisation of EBOV (Mayinga) GP pseudotyped (**a**) HIV-1 and (**b**) VSV by EVD survivor and negative plasma samples. The IC_50_ of pseudotyped virus neutralisation were estimated by model of nonlinear regression dose–response curves. Data are shown for individuals and the geometric mean with 95% confidence interval (95% CI). Dotted lines represent background level of neutralisation. Background level of pseudotyped HIV-1 neutralisation (IC_50_ 6.28 reciprocal dilution) is equal to UK negative control plasma mean plus two standard deviations, n = 7. Background level of pseudotyped VSV neutralisation is equal to the lowest dilution of sample tested in the assay (1/20). Statistically significant differences are highlighted (**p < 0.01, ****p < 0.0001; Mann–Whitney).

In order to compare the specificity and sensitivity of EBOV GP pseudotyped HIV-1 and VSV-based assays, we evaluated neutralisation of the EBOV GP pseudotyped HIV-1 and VSV using plasma samples collected from 30 EVD survivors of the 2013–2016 EBOV outbreak and 10 negative control donors from Guinea. The IC_50_ of pseudotyped virus neutralisation were estimated by model of nonlinear regression dose–response curves (Supplementary Table S2). Neutralisation of EBOV GP pseudotyped HIV-1 and VSV by positive (EVD survivor) and negative (UK donor) control plasma was assessed in several independent assays (Supplementary Fig. S2). The background level of neutralisation was determined using plasma from a UK negative control donor. For the HIV-1-based assay this was calculated as IC_50_ 6.28 reciprocal dilution. The negative control plasma displayed no neutralising activity against EBOV GP pseudotyped VSV, and therefore the background level of neutralisation was assigned the lowest dilution of sample tested in the assay (1/20). In the HIV-1-based assay, dose–response curves were unable to be fitted for three of the 30 EVD survivor samples and six of the samples were deemed below the background level of neutralisation. In contrast, a dose–response curve was unable to be fitted for only one of the EVD survivor samples tested in the VSV-based neutralisation assay. In the HIV-1-based assay, three of the 10 negative plasma samples tested were above the background level of neutralisation, whereas only one of the negative samples tested was above the background level of neutralisation in the VSV-based assay. Although some differences in the discriminatory power of positive and negative samples between the assays were observed, a statistically significant difference in neutralisation titres was detected between the EVD survivors and negative plasma samples in the HIV-1-based assay (Mann–Whitney, *p* = 0.0054) (Fig. 4a) and in the VSV-based assay (Mann–Whitney, *p* < 0.0001) (Fig. 4b, Supplementary Table S3). Remarkably, this difference was more significant and the separation of the positive and negative plasma was better in the VSV-based assay (Fig. 4b). The sum of these results clearly show that the VSV-based EBOV GP neutralisation assay displayed better reliability, specificity and sensitivity compared to the HIV-1-based assay.

### Correlation with live EBOV neutralisation

**Figure 5:**
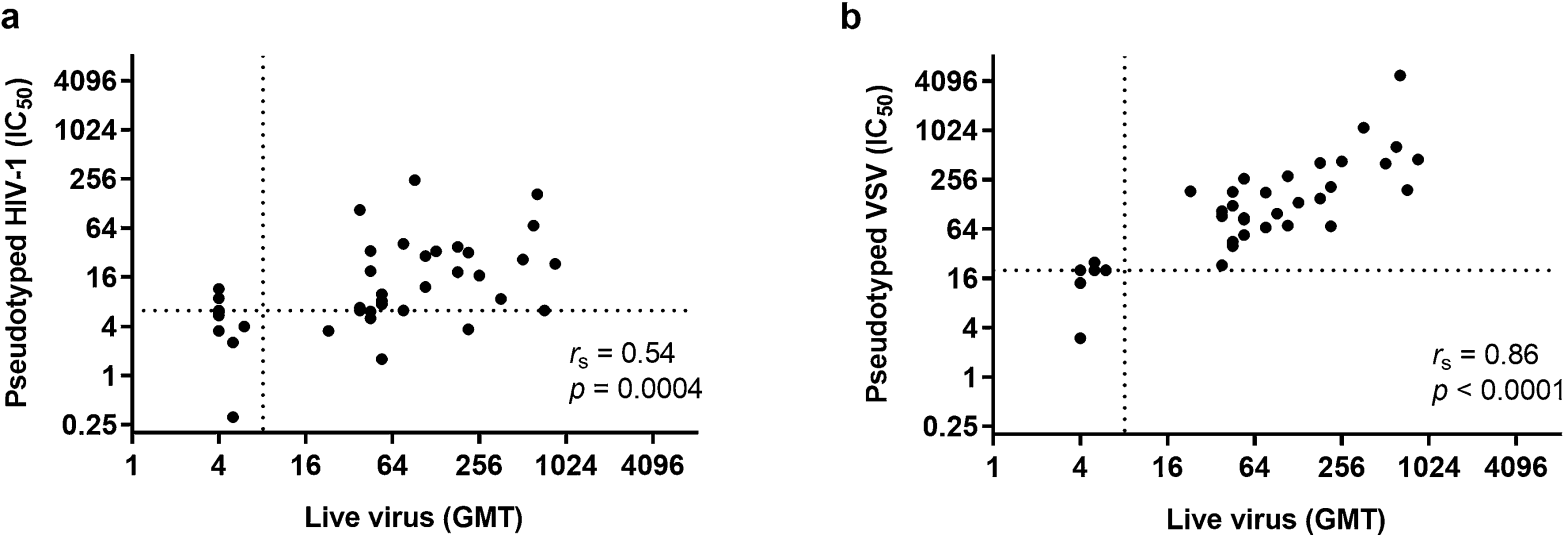
Correlation of EBOV GP pseudotyped (**a**) HIV-1 and (**b**) VSV (IC_50_) with live EBOV (GMT) neutralisation using the nonparametric Spearman correlation coefficient, n = 40. Dotted lines represent background level of neutralisation. Background level of pseudotyped HIV-1 neutralisation (IC_50_ 6.28 reciprocal dilution) is equal to UK negative control plasma mean plus two standard deviations, n = 7. Background level of pseudotyped VSV neutralisation is equal to the lowest dilution of sample tested in the assay (1/20). Seropositivity in the live EBOV neutralisation assay is defined by a GMT > 8.

The neutralising capacity of the individual plasma samples against authentic EBOV was assessed by a live virus neutralisation assay (Supplementary Table S3, Supplementary Fig. S3). When IC_50_ values of EBOV GP pseudotyped HIV-1 neutralisation of the 30 EVD survivor and 10 negative plasma samples were compared with GMT values for the live EBOV neutralisation assay, a positive correlation (*r*_s_ = 0.54) was determined using the nonparametric Spearman correlation coefficient (Fig. 5a) and this was statistically significant (*p* = 0.0004). Remarkably, a stronger statistically significant (*p* < 0.0001) positive correlation (*r*_s_ = 0.86) was observed when IC_50_ values of EBOV GP pseudotyped VSV neutralisation were compared with GMT values for the live EBOV neutralisation assay (Fig. 5b). The correlation coefficients for EBOV GP HIV-1 and VSV IC_50_ compared with live EBOV GMT without the negative controls were 0.38 (*p* = 0.0375) and 0.69 (*p* < 0.0001), respectively. Therefore, the VSV-based EBOV GP pseudotyped virus neutralisation assay correlated better with live EBOV neutralisation than the HIV-1-based neutralisation assay.

## Discussion

Pseudotyped viruses can be used as alternatives to infectious virus in serological assays to measure neutralising antibodies to viral envelope glycoproteins^11^. Pseudotyped virus assays used to profile neutralising antibody responses against severe acute respiratory syndrome-associated coronavirus (SARS-CoV)^26^, influenza (H5N1 and H7N9)^27–29^, rabies^30,31^ and chikungunya virus^32^, for example, found that results correlated well with those from replication-competent or live virus assays. A high degree of correlation has been demonstrated between EBOV CL4 PRNT and an EBOV pseudotyped VSV CL2 fluorescence reduction neutralisation test (FRNT)^33^. However, pseudotyped virus assays may not always accurately determine neutralisation^34,35^. Live EBOV and EBOV GP pseudotyped neutralisation assays have previously been shown to yield variable results^8,36^, which could be due to differing experimental conditions and viral systems. It is therefore important to optimise pseudotyped virus neutralisation assays in context of the particular viral GP being studied in order to obtain reliable specificity and sensitivity. The aim of this study was to assess the suitability of EBOV GP pseudotyped HIV-1 and VSV systems to measure the neutralising ability of plasma from EVD survivors, when compared to live EBOV neutralisation.

Reporter activity was detected in all cell lines (293T/17, Huh-7, HeLa and Vero E6) infected with EBOV GP (Mayinga) pseudotyped HIV-1 and VSV, demonstrating the broad tissue range conferred by EBOV GP, although differences in luminescence were observed. This may reflect general defects in viral entry in different cells. A relatively lower level of EBOV GP pseudotyped HIV-1 transduction was exhibited by Vero E6 cells, which might be due to an intrinsic restriction factor, TRIM5α, which restricts retroviral infection by specifically recognising the HIV-1 capsid and promoting its rapid, premature disassembly^37^. Highest TCID_50_ values were obtained following EBOV GP pseudotyped HIV-1 and VSV infection of 293T/17 and Vero E6 cells, respectively. There seemed to be large variability of the luminescent measurement for the VSV-based platform, which may be caused by the sensitive nature of the luciferase signal detection. This highlights the importance of titrating each pseudotyped virus batch before use in neutralisation assays, and the inclusion of multiple replicates.

The EBOV GP pseudotyped viruses were used to assess the neutralising activity of a human anti-EBOV GP mAb, KZ52. KZ52 has been shown previously to neutralise EBOV pseudotyped viruses^17,19,38^. However, within the range tested here, KZ52 did not display neutralisation against EBOV GP pseudotyped HIV-1, suggesting that the EBOV GP on the pseudotyped HIV-1 might be at higher levels, thereby reducing assay sensitivity, and neutralisation may be observed using a higher concentration of KZ52. In contrast, KZ52 was able to neutralise the EBOV GP pseudotyped VSV.

To assess the effects of differing amounts of pseudotyped virus input on neutralisation, plasma from an EVD survivor of the 2013–2016 EBOV outbreak and KZ52 were screened against different amounts of the EBOV GP pseudotyped HIV-1 and VSV, respectively. Decreasing quantities of pseudotyped HIV-1 led to more variable and unreliable results, and the KZ52 IC_50_ of pseudotyped virus neutralisation decreased with decreasing amounts of EBOV GP pseudotyped VSV input. The variability in neutralisation observed between different amounts of pseudotyped virus input highlights the importance of including standards or reference material with a known activity or potency when comparing neutralising activity, allowing calibration of results^39^.

Both pseudotyped virus systems were able to measure neutralising antibodies in plasma from EVD convalescent patients, and results correlated positively with a live EBOV neutralisation assay. However, the discriminatory power of the HIV-1-based assay with regards to differing antibody titres appeared to be low. Some of the samples tested, which showed neutralising activity against live EBOV, did not display neutralisation against the pseudotyped virus and vice versa, therefore raising questions on the sensitivity and specificity of the pseudotyped HIV-1 assay.

In the current study, human embryonic kidney (293T/17) cells were used for the pseudotyped HIV-1 neutralisation assays, whereas African green monkey kidney (Vero) cells were used in the VSV-based assay and also the live EBOV assay. Therefore, this could account for some of the differences in results observed between the two assays and for the better performance of the VSV-based assay in relation to live EBOV neutralisation. Also, the HIV-1- and VSV-based pseudotyped virus systems assessed in the current study utilise different transfection methods, which could have implications on the composition of the pseudotyped viruses, density and/or glycosylation of the viral envelope protein on the surface, and consequently neutralisation results. This highlights the importance of assessing experimental conditions and methodology when developing and optimising pseudotyped virus neutralisation assays. A limitation to this study was that the level of EBOV GP incorporation per pseudotyped virus type could not be assessed. Also, for the VSV-based pseudotyped virus system, traces of VSV-G from the rVSV-ΔG-Luc-VSV-G virus could be recycled into newly pseudotyped virions^40^. Therefore, the use of anti-VSV-G hybridoma cell culture supernatant could give rise to pseudotyped virions covered by anti-VSV-G antibodies, but are still infectious due to Ebola GP. This could potentially induce plasma specific reactivity of virions due to bound anti-VSV-G antibodies more than EBOV GP specific reactivity.

There are several differences between EBOV GP pseudotyped and live EBOV neutralisation assays that could affect their results^8^. Due to their non-replicating nature, such pseudotype systems do not recapitulate all steps in the viral life cycle that may potentially be targeted by neutralising antibodies^41^. In addition, the round, spherical shape of EBOV GP pseudotyped HIV-1 or bullet shape of EBOV GP pseudotyped VSV compared to the filamentous shape of authentic EBOV could affect their susceptibility to neutralisation. Also, the density of GP on the surface of the pseudotyped virus may not be the same as that found on live EBOV and may result in the loss or masking of quaternary epitopes^11,42^. Furthermore, GP maturation and assembly in live EBOV could be different in the generation of an EBOV pseudotyped virus and may result in different targets and/or conformational epitopes when using whole live EBOV as opposed to EBOV GP alone in a pseudotyped virus. The presence of shed GP or secreted GP (sGP) in the live EBOV assay compared to absence in the EBOV GP pseudotyped virus assays could also have an effect on neutralisation. In the live EBOV assay, shed GP and sGP could reduce neutralisation of circulating virus by cross-reactive antibodies to surface GP. However, in the current study, weaker relative neutralisation was observed in the HIV-1 based pseudotyped virus assay. Therefore, it is possible that cell debris or free GP generated during EBOV GP pseudotyped HIV-1 production by polyethylenimine (PEI) transfection could be interfering with neutralisation. Finally, detection of infected cells via measurement of luminescence in the EBOV GP pseudotyped virus neutralisation assay compared to plaque formation in the live EBOV neutralisation assay could affect neutralisation readout.

EBOV GP pseudotyped virus neutralisation assays have value for vaccine evaluation and assessment of convalescent blood products and mAbs for use as immunotherapeutics. However, pseudotyped virus assays may not always accurately determine neutralisation when compared with neutralisation against live virus. In this study, both EBOV GP pseudotyped HIV-1 and VSV assays were able to detect neutralisation of plasma from EVD survivors and correlated positively with live EBOV neutralisation. However, the VSV-based assay performed better than the HIV-1-based assay in relation to specificity, sensitivity, and correlation with the live EBOV neutralisation assay. This research has highlighted the importance of optimising pseudotyped virus neutralisation assays in context of the particular viral GP being studied, especially when evaluating vaccine responses and therapeutics, and could provide a better understanding of the correlates of protection against EBOV.

## Methods

### Plasmids and cells

The HIV-1 *gag-pol* plasmid p8.91^43^, the firefly luciferase reporter construct pCSFLW^44^ and a pCAGGS EBOV (Mayinga) GP (GenBank accession number NC_002549) expression construct were kind gifts from Edward Wright[University of Sussex, Brighton, United Kingdom (UK)].

Human embryonic kidney (HEK) 293T clone 17 cells (293T/17; American Type Culture Collection (ATCC), Teddington, UK, CRL-11268) were used for all transfections and as a target cell line for titration and pseudotyped HIV-1 neutralisation assays. Vero E6[Vero 76, clone E6, Vero E6 (European Culture of Authenticated Cell Cultures (ECACC), Salisbury, UK, 85020206) and Huh-7 (Arvind Patel, University of Glasgow, UK) cells were used as target cell lines. All cell lines were cultured at 5% CO_2_ in Dulbecco’s Modified Eagle Medium (DMEM), high glucose, with l-glutamine (Gibco, Paisley, UK) supplemented with 10% fetal bovine serum (FBS), heat inactivated (Sigma-Aldrich, Gillingham, UK). HeLa cells (ECACC 93021013) were also used as a target cell line and were cultured in Minimum Essential Media (MEM) + GlutaMAX (Life Technologies, Paisley, UK) supplemented with 10% FBS and 1 × MEM Non-Essential Amino Acids (NEAA) solution (Life Technologies).

### Human samples and purified human antibody

Plasma samples from EVD survivors of the 2013–2016 EBOV outbreak recruited 3 to 14 months post-infection from two regions of Guinea (Guéckédou and Coyah) and from negative control blood donors in the UK and Guinea, who were not knowingly exposed to persons with EVD and did not attend high risk events such as funerals, were heat inactivated at 56 °C for 30 min. The samples were obtained from a pre-existing biobank, for which live EBOV neutralisation^45^ data were available (Thomas Strecker, Philipps University Marburg, Germany) in link-anonymised format. All experiments involving live EBOV were performed in a CL4 facility at Philipps University Marburg, Germany. The biobank was established by Horizon 2020 EU research initiative ‘EVIDENT’. All experimental protocols used in this study were approved by the Guinean National Ethics Committee for Research and Health [Comité National d'Ethique pour la Recherche en Santé (CNERS)]. All methods were carried out in accordance with the relevant guidelines and regulations under ethical approval No. 33/CNERS/15. Informed consent was obtained from all participants.

Protein A purified human anti-EBOV GP mAb KZ52 (IBT Bioservices Rockville, Maryland (MD), USA) was also tested in the EBOV GP pseudotyped virus neutralisation assays.

### Production of pseudotyped viruses

The generation of HIV-1 pseudotyped viruses was performed as detailed previously^44,46,47^. Twenty-four hours prior to transfection, approximately 8 × 10^5^ 293T/17 cells were seeded into sterile, 6-well cell culture plates (Corning, Ewloe, UK) and incubated at 37 °C, 5% CO_2_ and 95% humidity until 60–80% confluence. The HIV *gag-pol* plasmid, p8.91, and the firefly luciferase reporter construct, pCSFLW, were transfected simultaneously with the EBOV (Mayinga) GP expression vector at a ratio of 0.6:0.9:0.6 µg (core:reporter:envelope) using 10 µl of 1 µg/ml polyethylenimine (PEI) (Sigma-Aldrich) per 1 µg DNA in Opti-MEM medium (Gibco). Following overnight transfection, the cells were incubated with fresh medium and incubated at 37 °C, 5% CO_2_. Pseudotyped virus supernatants were harvested at 48 and 72 h post-transfection, passed through a 0.45 µm pore filter (Millex, Millipore, Watford, UK) and stored at − 80 °C.

EBOV GP pseudotyped VSVs were prepared using recombinant VSV, in which the VSV-G gene had been deleted (rVSV-ΔG) and replaced with a luciferase reporter gene (rVSV-ΔG-Luc) by a method similar to that described previously^48^. Twenty-four hours prior to transfection, approximately 2.4 × 10^6^ 293T/17 cells were seeded into sterile, 100 mm cell culture dishes (Corning) and incubated at 37 °C, 5% CO_2_ and 95% humidity until 60–80% confluence. The cells were transfected with the EBOV GP expression vectors using *Trans*IT-LT1 Transfection Reagent (Mirus Bio, Madison, Wisconsin (WI), USA) as per the manufacturer’s instructions. Following overnight transfection, the medium was removed and the cells were infected with rVSV-ΔG-Luc that was pseudotyped with the VSV glycoprotein (rVSV-ΔG-Luc-VSV-G) (Masayuki Saijo, National Institute of Infectious Diseases, Tokyo, Japan) at a multiplicity of infection (MOI) of 5 in Opti-MEM medium and incubated at 37 °C, 5% CO_2_. After 2 h, the inoculum was removed, cells were washed twice with Dulbecco’s phosphate buffered saline (DPBS) (Gibco) and fresh medium was added. Pseudotyped virus supernatants were harvested at 18–24 h post-infection, clarified twice by centrifugation at 200x*g* for 5 min at 10 °C and stored at − 80 °C. Prior to use, the pseudotyped viruses were incubated with anti-VSV-G hybridoma cell culture supernatant (Masayuki Saijo, National Institute of Infectious Diseases, Tokyo, Japan) at a 1:125 dilution for 1 h at 37 °C to reduce background infection mediated by residual virus possessing VSV-G, which can be carried over during preparation^48^.

All experiments involving pseudotyped viruses were performed in a CL2 facility at Public Health England (PHE), Porton Down, UK.

### Pseudotyped virus titration and neutralisation assays

Titration and neutralisation assays were performed in 96-well solid white flat bottom polystyrene TC-treated microplates (Corning) and were based upon previously described protocols^44,46,48^.

For pseudotyped HIV-1 titration assays, five-fold serial dilutions of pseudotyped virus at a starting dilution of 1:5 were prepared in quadruplicate in Opti-MEM medium at a final volume of 100 µl/well. 100 µl of approximately 2 × 10^4^ 293T/17, Huh-7 or Vero E6 cells, or 1 × 10^4^ HeLa cells were then added to each well and incubated at 37 °C, 5% CO_2_ for 48 h. The medium was removed and 50 µl of a 50:50 mix of Bright-Glo luciferase assay reagent (Promega, Southampton, UK):fresh medium was added to each well and incubated for at least 2 min at room temperature to allow complete cell lysis. Luminescence was measured using a Glomax-Multi + detection system luminometer (Promega) and relative luminescence units per ml (RLU/ml) were determined. The negative cut-off was set at 2.5 times the average RLUs of the cells only control wells. 50% tissue culture infectious dose (TCID_50_)/ml values were determined using the Reed-Muench method^49^.

For the pseudotyped HIV-1 neutralisation assay, two or threefold serial dilutions of plasma samples at a starting dilution of 1:5 or 1:10, respectively, were prepared in duplicate in Opti-MEM medium at a final volume of 50 µl/well and incubated with 50 µl of a standardised RLU per well of pseudotyped virus (as calculated from the titration assay), prepared in Opti-MEM medium, for 1 h at 37 °C. 100 µl of approximately 2 × 10^5^ 293T/17 cells were then added to each well and incubated for 48 h at 37 °C, 5% CO_2_, prior to taking a chemiluminescent readout as described above. Infectivity was calculated using the formula: Percentage (%) infectivity = [(RLU with sample)/(RLU without sample)] × 100.

For pseudotyped VSV titration assays, 24 h prior, approximately 2.5 × 10^4^ 293T/17 or 1 × 10^4^ Huh-7, HeLa cells or Vero E6 cells were seeded in 96-well microplates and incubated at 37 °C, 5% CO_2_ and 95% humidity. The medium was removed and two-fold serial dilutions of pseudotyped virus in Opti-MEM medium, starting with neat pseudotyped virus were added to each well in quadruplicate at a final volume of 100 µl/well. After 24 h, a chemiluminescent readout was taken and TCID_50_/ml values were determined as described above.

Twenty-four hours prior to pseudotyped VSV neutralisation, approximately 1 × 10^4^ Vero E6 cells were seeded and incubated as for titration above. Twofold serial dilutions of plasma samples at a starting dilution of 1:10 were prepared in duplicate in Opti-MEM medium at a final volume of 120 µl/well in 96-well microplates, and incubated with 120 µl of a standardised RLU per well of pseudotyped virus (as calculated from the titration assay), prepared in Opti-MEM medium, for 1 h at 37 °C. The medium was removed from the cells, 50 µl of the plasma-pseudotyped virus mixtures were added to each well in quadruplicate at incubated at 37 °C, 5% CO_2_. After 1 h, 50 µl of fresh medium was added to each well. Luminescence was measured after 24 h and infectivity was calculated as described above.

### Statistical analysis

Pseudotyped virus neutralisation assay raw data were normalised as percentage (%) infection relative to mean values for pseudotyped virus only controls (equivalent to 100% infection), then IC_50_ of pseudotyped virus neutralisation were estimated by model of nonlinear regression fit with settings for log (inhibitor) vs. normalised response curves using GraphPad Prism v5 (San Diego, California (CA), USA).

Statistical comparison between two unpaired groups was performed using the Mann–Whitney test (GraphPad Prism v5). Correlation between two variables was quantified using Spearman nonparametric correlation (GraphPad Prism v5).

## Supplementary information

Supplementary Information.
