# Supplementary material for: Pseudotyping of VSV with Ebola virus glycoprotein is superior to HIV-1 for the assessment of neutralising antibodies
Source: Sci Rep. 2020 Aug 31;10:14289. doi: 10.1038/s41598-020-71225-1 (PMC7459353; doi:10.1038/s41598-020-71225-1)
Supplement: Supplementary file 1 — Supplementary Information. [file 41598_2020_71225_MOESM1_ESM.pdf]

Supplementary Information

Pseudotyping of VSV with Ebola virus glycoprotein is superior to HIV-1 for the assessment of neutralising antibodies

Kimberley Steeds[1], Yper Hall[1], Gillian S. Slack[1], Stephanie Longet[1], Thomas Strecker[2], Sarah Katharina Fehling[2], Edward Wright[3], Joseph Akoi Bore[4], Fara Raymond Koundouno[5], Mandy Kader Konde[6], Roger Hewson[1], Julian A. Hiscox[7], Georgios Pollakis[7] & Miles W. Carroll[1,*]

[1]Public Health England (PHE), Porton Down, Salisbury, Wiltshire, UK.

[2]Institute of Virology, Philipps University Marburg, Marburg, Germany.

[3]School of Life Sciences, University of Sussex, Brighton, UK.

[4]Institut National de Santé Publique, Conakry, Republic of Guinea.

[5]University Julius Nyerere of Kankan, Conakry, Republic of Guinea.

[6]Centre d'Excellence de Formation et Recherche sur le Paludisme et les Maladies Prioritaires en Guinée (CEFORPAG), Ratoma, Conakry, Republic of Guinea.

[7]Institute of Infection and Global Health (IGH), University of Liverpool, Liverpool, UK.

*Corresponding author.

miles.carroll@phe.gov.uk

Background luminescence of cells only controls

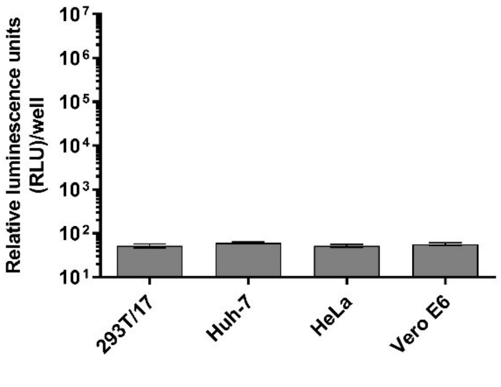

Supplementary Figure S1. Background luminescence of 293T/17, Huh-7, HeLa and Vero E6 cell lines. Error bars are one standard error above and below the mean, n = 8.

Assessment of EBOV GP pseudotyped virus input for neutralisation

Supplementary Table S1. Effect of EBOV (Mayinga) GP pseudotyped VSV input on neutralisation by anti-EBOV GP mAb, KZ52. Percentage infectivity was calculated relative to pseudotyped virus only controls, and IC_{50} of EBOV GP pseudotyped VSV neutralisation were estimated by model of nonlinear regression dose-response curves.

EBOV GP pseudotyped VSV input (RLU/ml)	KZ52 IC_{50} (µg/ml)	95% CI
6.2×10^4	0.08	0.04, 0.17
3.9×10^4	0.07	0.03, 0.16
3.3×10^4	0.03	0.02, 0.05
2.0×10^4	0.01	0.01, 0.03

Neutralisation of EBOV GP pseudotyped viruses by EVD survivor plasma

Supplementary Table S2. Ebola virus disease (EVD) survivor samples tested in the EBOV GP pseudotyped virus neutralisation assays. Plasma samples from EVD survivors of the 2013-2016 EBOV outbreak were obtained from a pre-existing biobank. Live EBOV (Mayinga) neutralisation data were available for each sample. IC = Inhibitory concentration (reciprocal dilution), GMT = Geometric mean titre.

Sample	EBOV GP pseudotyped virus neutralisation				Live EBOV neutralisation
	HIV-1		VSV		
	IC_{50}	IC_{80}	IC_{50}	IC_{80}	GMT
G041	23.38	5.85	452.69	113.17	861
G048	6.28	1.57	191.72	47.93	724
G011	166.39	41.60	4787.00	1196.75	645
G037	68.73	17.18	645.00	161.25	609
G036	26.29	6.57	404.00	101.00	512
CS090	8.74	2.19	1106.00	276.50	362
CS053	16.76	4.19	959.00	239.75	256
G021	32.00	8.00	208.94	52.24	215
G035	3.70	0.93	69.00	17.25	215
G001	37.52	9.38	150.49	37.62	181
G014	18.47	4.62	408.00	102.00	181
G005	33.10	8.28	134.00	33.50	128
G045	12.25	3.06	70.18	17.55	108
G013	28.94	7.24	281.69	70.42	108
G025	246.85	61.71	98.81	24.70	91
G024	6.28	1.57	67.00	16.75	76
G044	40.85	10.21	179.00	44.75	76
G033	7.49	1.87	87.03	21.76	54
G028	1.60	0.40	263.00	65.75	54
G031	8.29	2.07	83.00	20.75	54
G018	9.93	2.48	52.00	13.00	54
G026	5.03	1.26	39.53	9.88	45
G038	33.46	8.37	123.00	30.75	45
G040	18.98	4.75	45.00	11.25	45
G020	6.10	1.53	182.00	45.50	45
G027	6.81	1.70	92.00	23.00	38
G019	6.28	1.57	23.00	5.75	38
CS084	106.72	26.68	106.00	26.50	38
G030	3.54	0.89	185.00	46.25	23
G022	4.01	1.00	20.00	5.00	6

Reproducibility of EBOV GP pseudotyped virus neutralisation

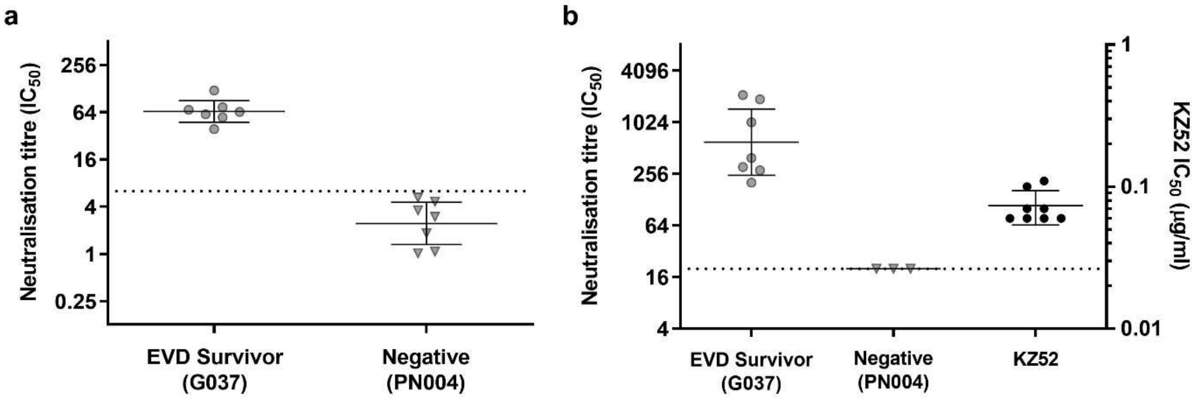

Supplementary Figure S2. Reproducibility of EBOV (Mayinga) GP pseudotyped (a) HIV-1 and (b) VSV neutralisation by control samples. The IC_{50} of pseudotyped virus neutralisation were estimated by model of nonlinear regression dose-response curves. Data are shown for individuals and the geometric mean with 95% CI. Dotted lines represent background level of neutralisation. Background level of pseudotyped HIV-1 neutralisation (IC_{50} 6.28 reciprocal dilution) is equal to UK negative control plasma mean plus two standard deviations, n = 7. Background level of pseudotyped VSV neutralisation is equal to the lowest dilution of sample tested in the assay (1/20).

Neutralisation of EBOV GP pseudotyped virus and live EBOV by EVD survivor plasma

Supplementary Table S3. Differences in neutralisation of EBOV GP pseudotyped virus and live EBOV by EVD survivor and negative plasma samples.

Mann-Whitney test		*p* value	U	Difference between medians (95% CI)
**EBOV GP pseudotyped virus**	**HIV-1**	0.0054	60.5	8.6 (1.3, 25.4)
	**VSV**	< 0.0001	5.5	128 (66, 243)
**Live EBOV**		< 0.0001	0.0	87 (49, 211)

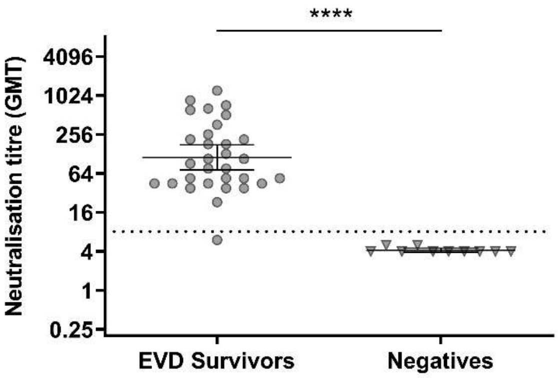

Supplementary Figure S3. Neutralisation of live EBOV (Mayinga) by EVD survivor and negative plasma samples. Data are shown for individuals and the geometric mean with 95% CI. Neutralisation tires were calculated as geometric mean titres (GMT) of four replicates. Dotted line represents background level of neutralisation. Seropositivity is defined by a GMT > 8. Statistically significant difference is highlighted (*****p* < 0.0001; Mann-Whitney).
